# EPS8L3 promotes pancreatic cancer proliferation and metastasis by activating GSK3B

**DOI:** 10.5937/jomb0-38840

**Published:** 2023-01-20

**Authors:** Zun Fan, Ming Li, Yinjie Xu, Chenxing Ge, Jianfeng Gu

**Affiliations:** 1 Changshu No.1 People's Hospital Affiliated to Soochow University, Department of General Surgery, Changshu, China

**Keywords:** EPS8L3, GSK3B, pancreatic cancer, proliferation, metastasi, EPS8L3, GSK3B, kancer pankreasa, proliferacija, metastaza

## Abstract

**Background:**

We intended to investigate the role and regulatory mechanism of EPS8L3 in increase the development of pancreatic cancer (PC).

**Methods:**

In order to analyze the relationship between EPS8L3 level and clinicopathological indicators of PC patients, qRT-PCR was used to detect the expression of EPS8L3 in tumor specimens of 40 PC patients. EPS8L3 knockdown models were then constructed in PC cell lines. Furthermore, the effect of EPS8L3 on PC cell function was analyzed by CCK-8 and Transwell assay. Dual luciferase reporter gene assay and recovery assay were used to further investigate the underlying mechanism.

**Results:**

qRT-PCR results indicated that EPS8L3 was highly expressed in PC tissues compared with adjacent ones. At the same time, the incidence of distant metastasis was higher in PC patients with high EPS8L3 level.* In vitro* analysis such as CCK-8 and Transwell experimentations indicated that knockdown of EPS8L3 markedly inhibited the proliferative and metastatic ability. Bio-informatics together with luciferase report assay proposing that EPS8L3 can target GSK3B. Western Blot results revealed that knockdown of EPS8L3 markedly reduced the GSK3B expression in PC cells, and there was a positively associated between the two in PC cells. In addition, the recovery experimentation proved that EPS8L3 and GSK3B have a mutual regulation effect. Overexpression of GSK3B can reversal the prohibitive effect of EPS8L3 knockdown on the malignant development of PC cells, thereby jointly regulating the occurrence and development of PC.

**Conclusions:**

EPS8L3 promotes the development of PC by regulating GSK3B, suggesting that EPS8L3 can be used as a biomarker for early diagnosis and treatment of PC.

## Abstract (sr)

**Uvod:**

Nameravali smo da istražimo ulogu i regulatorni mehanizam EPS8L3 u povećanju razvoja raka pankreasa (PC).

**Metode:**

U cilju analize odnosa između nivoa EPS8L3 i kliničkopatoloških indikatora pacijenata sa PC, kRT-PCR je korišćen za detekciju ekspresije EPS8L3 u uzorcima tumora 40 pacijenata sa PC. EPS8L3 modeli obaranja su zatim konstruisani u PC ćelijskim linijama. Staviše, efekat EPS8L3 na funkciju PC ćelija analiziran je CCK-8 i Transvell testom. Dualni test reporter gena luciferaze i test oporavka korišćeni su za dalje istraživanje osnovnog mehanizma.

## Introduction

Recently, with the continuous increase of the population base, the aging of the population has become increasingly serious, and the social demographic structure has changed dramatically, leading to the increasing incidence and mortality of cancer. Cancer has become a major public health problem affecting human life and health [1] [2] [3]. Currently, Pancreatic cancer (PC) is one of the most frequent and malignant tumors in the digestive system. The median survival time is about 3–6 months, and the 5-year survival rate is less than 5% [4] [5]. According to the statistical analysis of the latest epidemiological survey, PC has an extremely high mortality rate in malignant tumors. There are millions of patients who die of PC every year in the world, and its mortality rate ranks fourth [4] [5] [6]. Although we have been constantly exploring the diagnosis along with the treatment of PC in the past several decades, the overall survival of PC is still extremely poor. Many patients are diagnosed in the middle and late stages, and are accompanied by local invasion or distant metastasis. Only about 20% of patients can undergo surgery [7] [8]. Therefore, exploring molecular targets that can specifically regulate the occurrence and development of PC and elucidate their molecular mechanisms has important theoretical guiding significance and clinical application value for improving early diagnosis and clinical treatment of PC [9] [10].

Epidermal growth factor receptor pathway substrate 8 (EPS8) is an intracellular signaling pathwaysubstrate. Located on chromosome 12q23.q24, it contains approximately 821 amino acids with a relative molecular weight of 97 kD [11] [12]. Some studies have found that EPS8 has three gene analog members, namely EPS8L1, EPS8L2, and EPS8L3, which have the same linear topological structure [12] [13]. Among them, EPS8L3 can participate in tumor development, actin cytoskeleton reorganization, filamentous actin microfilament aggregation via multiple signaling pathways [12] [14] [15]. Therefore, in this study, by collecting PC tissue samples, we analyzed the relationship between EPS8L3 level and clinical characteristics of PC, and further explored the specific molecular mechanism of EPS8L3 mediated PC malignant development, aiming at providing experimental basis for its clinical application.

## Materials and methods

### PC samples collection

Forty pairs of pancreatic tissues were resected from surgical patients from our hospital. All patients had not received medical treatment and/or radiotherapy before surgery. The excised experimental specimens were immediately stored at -80°C. Two experienced pathologists diagnose all tissue samples used in the experimentation. Each part of this experimentation conformed to ethical requirements; and all participants provided the signed informed consents.

### Cells culture

PC cell lines AsPC-1, PANC-1, CFPAC-1, MIA PaCa-2, BxPC-3 and normal pancreatic ductal epithelial cell line HPNE were all purchased from the ATCC (Manassas, VA, USA). All cell lines were cultured inDMEM high glucose medium containing 10% FBS (Gibco, Rockville, MD, USA), penicillin (100 U/mL) and streptomycin (100 μg/mL) in a 37°C, 5% CO_2_ incubator. Detection of migrative ability was determined via Transwell assay.

### Transfection

EPS8L3 knockdown lentiviral vector was designed, constructed and packaged by Shanghai Jima Pharmaceutical Technology Co., Ltd. (Shanghai, China). Subsequently, lentivirus transfection was performed following instructions.

### Cell proliferation assays

48 h after transfection, 2000 cells were seeded into each 96-well. After culturing the cells for 24, 48, 72 and 96 hours, 10 μL of CCK-8 (Dojindo Laboratories, Kumamoto, Japan) reagent was added, and the culture continued for 2 hours. Then the OD value of each well at 450 nm absorption wavelength was measured using a microplate reader.

### Real-time fluorescent quantitative PCR

Total RNA was extracted using TRIzol kit and reverse transcribed into cDNA. QRT-PCR reaction was performed using SYBR on the Applied Biosystems Step One Plus real-time PCR instrument.Primer sequences used in this study were listed below (5’-3’): EPS8L3: F: CTCCATCCTGTCCATCACCG, R: AGTCGAGGTCTGCTTTGCTC; GSK3B: F: ACAGCAGCGTCAGATGCTAA, R: AACGTGACCAGTGTTGCTGA; β-actin: F: CCTGGCACCCAGCACAAT, R: TGCCGTAGGTGTCCCTTTG.

### Dual luciferase reporter assay

HEK293T cells were seeded in 24-well plates and co-transfected with pcDNA-NC/pcDNA-GSK3B and wild-type/mutant EPS8L3 pMIR luciferase reporter plasmids. 48 hours after transfection, transfected cells were lysed using the luciferase lysate. Then the dual luciferase reporter gene assay was performed following the instructions of the dual luciferase reporter gene system (Promega, Madison, WI, USA).

### Statistically analysis

All data analysis was processed using GraphPad V5.01 software (La Jolla, CA, USA). Student’s-t-test and one-way ANOVA was used to analyze the statistical difference between two groups and multiple groups. P values < 0.05 indicated significant difference.

## Results

### EPS8L3 was highly-expressed in PC tissues

**Figure 1:**
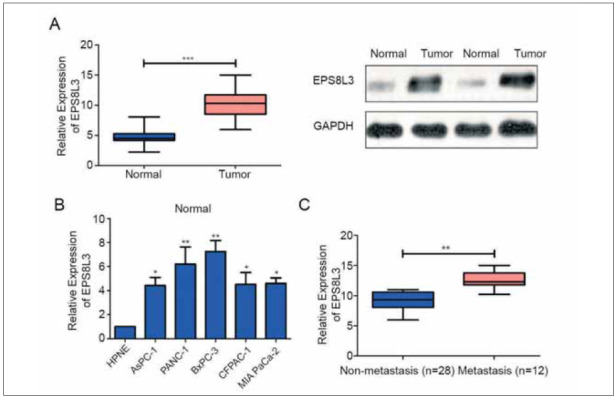
EPS8L3 is highly expressed in pancreatic cancer tissues and cell lines. (A) The expression of EPS8L3 in the tumor tissue of pancreatic cancer patients was significantly higher than that in the adjacent tissue; (B) the expressions of EPS8L3 pancreatic cancer cell lines PANC-1 and BxPC-3 were significantly increased; (C) the high expression of EPS8L3 was significantly correlated with the expression of EPS8L3 in patients with pancreatic cancer. The incidence of distant metastasis was positively correlated.

The results indicated that the level of EPS8L3 in tumor tissues of PC patients was significantly higher than that in adjacent tissues (Figure 1A), suggesting that EPS8L3 may play the role of oncogenes in PC. At the same time, in vitro qRT-PCR results also indicated that EPS8L3 was up-regulated in PC cell lines, especially in PANC-1 and BxPC-3 (Figure 1B). According to the qRT-PCR results of EPS8L3 level in 40 pairs of PC patient tissues, EPS8L3 level was divided into two groups, low expression group and high expression group, and chi-square test was used to analyze the correlation between EPS8L3 level and clinicopathological parameters of PC patients. As shown in Table 1, high expression of EPS8L3 and the incidence of distant metastasis in patients with PC showed a positive correlation (Figure 1C).

**Table 1:** Baseline data of the enrolled patients with pancreatic cancer.

Parameters	Number<br>of cases	EPS8L3 expression		P -value
		Low<br>(n=20)	High<br>(n=20)	
Age (years)				0.206
<60	20	8	12	
≥60	20	12	8	
Gender				0.519
Male	24	13	11	
Female	16	7	9	
T stage				0.327
T1-T2	25	11	14	
T3-T4	15	9	6	
Lymph node				0.091
No	27	16	11	
Yes	13	4	9	
Distance				0.038
No	28	17	11	
Yes	12	3	9	

### Knockdown of EPS8L3 inhibited proliferative ability and metastasis of PC cells

**Figure 2:**
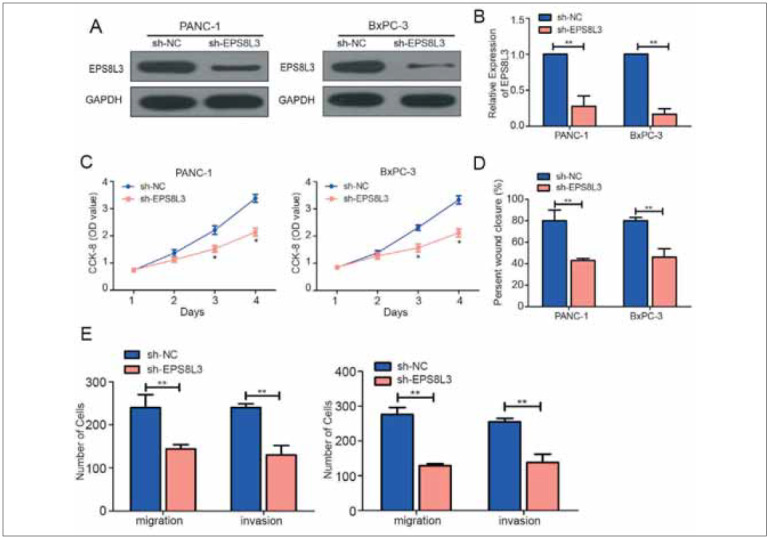
Silencing EPS8L3 inhibits the proliferation and metastatic ability of pancreatic cancer cells. (A) The protein level was significantly down-regulated after transfection of sh-EPS8L3 in pancreatic cancer cell lines PANC-1 and BxPC-3; (B) The mRNA level was significantly reduced after transfection of sh-EPS8L3 in pancreatic cancer cell lines PANC-1 and BxPC-3 down-regulated; (C) Pancreatic cancer cell lines PANC-1 and BxPC-3 transfected with sh-EPS8L3 significantly reduced cell proliferation; (D) Pancreatic cancer cell lines PANC-1 and BxPC-3 were transfected with sh-EPS8L3 The ability of cell migration was significantly attenuated; (E) The ability of cell invasion was significantly attenuated after transfection with sh-EPS8L3 in pancreatic cancer cell lines PANC-1 and BxPC-3.

To study the effect of EPS8L3 on PC, we established an EPS8L3-knockdown lentivirus model. After transfected of EPS8L3 knockdown vectors in PANC-1 and BxPC-3 cell lines, qRT-PCR and Western Blot substantiated their interference efficiency, indicating that the knockdown vector was successfully constructed (Figure 2A & Figure 2B). Subsequently, the CCK-8 experimentation revealed that the proliferative ability of PC cells were significantly weakened after EPS8L3 knockdown (Figure 2C). Moreover, the Transwell experimentations also suggested that knockdown of EPS8L3 markedly decreased metastasis of PC cells (Figure 2D and Figure 2E). These results indicated that EPS8L3 can markedly promote the PC cell proliferative ability and metastasis.

### EPS8L3 was bound to GSK3B

**Figure 3:**
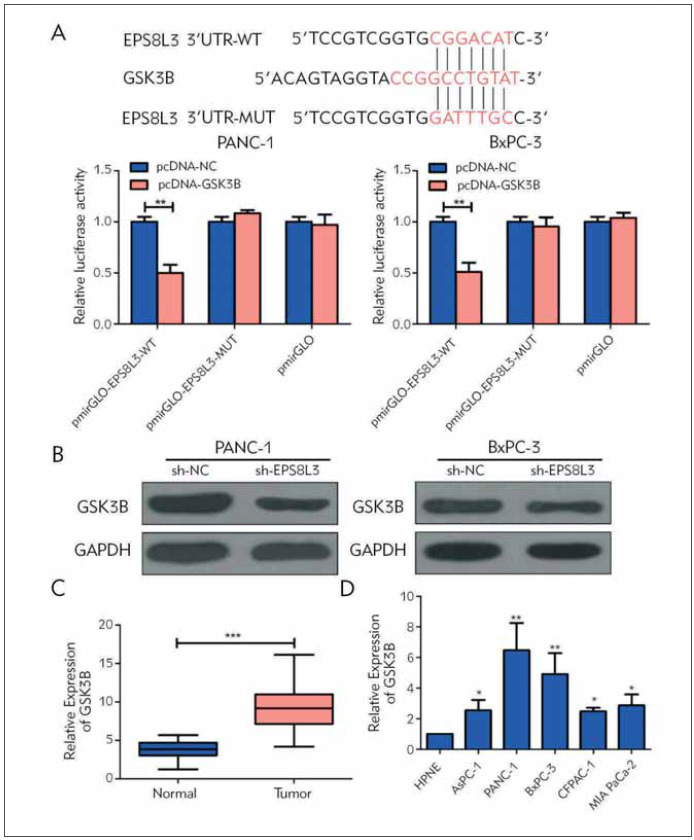
The targeting relationship between EPS8L3 and GSK3B. (A) The results of luciferase reporter gene experiments show that EPS8L3 can be targeted by GSK3B through specific binding sites; (B) In PANC-1 and BxPC-3 cell lines, knockdown of EPS8L3 can significantly reduce the expression level of GSK3B; ( C) The expression of GSK3B was also significantly higher in tumor tissues of pancreatic cancer patients than in adjacent tissues; (D) GSK3B was significantly overexpressed in pancreatic cancer cell lines.

To further verify the targeting effect of EPS8L3 on GSK3B, the wild-type and the mutant EPS8L3 sequence was cloned into the luciferase reporter plasmid pmirGLO, and co-transfected with GSK3B into the PC cells. Results indicated that EPS8L3 can target GSK3B by a specific binding site (Figure 3A). In addition, Western Blot experimentations suggested that knockdown of EPS8L3 can markedly reduce the GSK3B expression in the PC cells (Figure 3B). Moreover, qRT-PCR results suggested that the GSK3B level was markedly higher in PC tumor tissues as well as PC cell lines (Figure 3C & Figure 3D).

### EPS8L3 modulated GSK3B in PC cell lines

**Figure 4:**
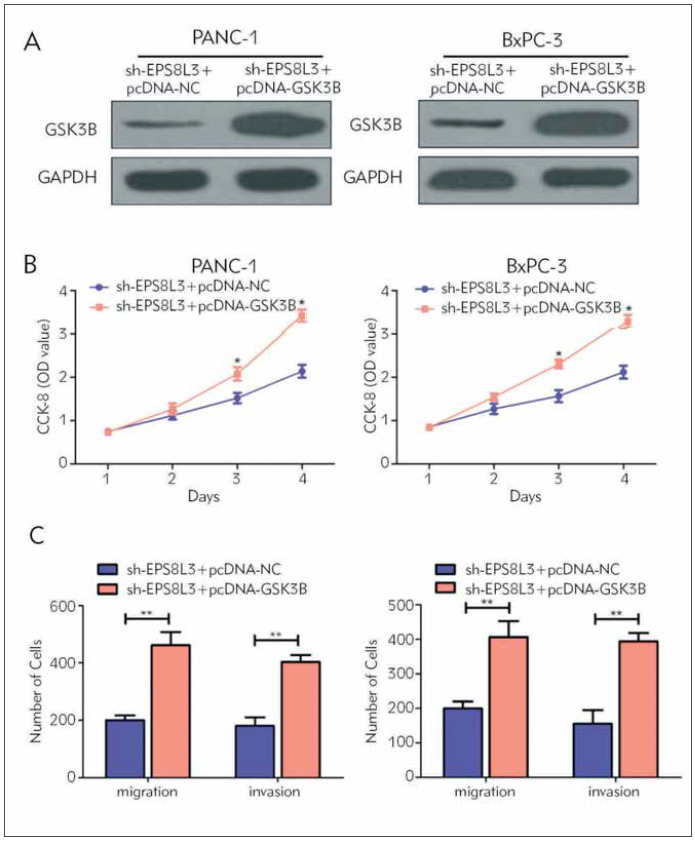
EPS8L3 promotes the malignant progression of pancreatic cancer by activating GSK3B. (A) GSK3B protein level was significantly up-regulated after co-transfection of EPS8L3 knockdown vector and GSK3B overexpression vector in pancreatic cancer cell lines PANC-1 and BxPC-3; (B) EPS8L3 knockdown by co-transfection of PANC-1 and BxPC-3 The proliferation ability of the vector and GSK3B overexpression vector was significantly increased; (C) PANC-1 and BxPC-3 co-transfected with EPS8L3 knockdown vector and GSK3B overexpression vector significantly increased the invasion and migration ability.

In order to further the regulation between EPS8L3 and GSK3B in PC, GSK3B was overexpressed in EPS8L3-knockdown PC cells. The Western Blot experimentation showed that GSK3B overexpression can markedly restored the expression of GSK3B (Figure 4A). Subsequently, the CCK-8 experimentation showed that GSK3B overexpression can reverse the inhibition of the proliferative ability of PC cells caused by EPS8L3 knockdown (Figure 4B). In addition, Transwell experimentations also suggested that GSK3B overexpression can reversal the reversal impact of EPS8L3-knockdown on the invasive and migrative ability of PC cells (Figure 4C). Therefore, we concluded that EPS8L3 may activate GSK3B, thereby promoting the proliferative ability and metastasis of PC cells.

## Discussion

Although progress has been made on the effective diagnosis as well as the treatment methods for PC, but the mortality rate of PC is still high, and its incidence and mortality rate are almost the same [4] [5] [6]. So far, there are no particularly effective drugs for PC; at the same time, less than 20% of PC patients are acceptable for surgery [7] [8]. The main reason is that the incidence of PC is hidden and the pathological process is rapid, and the patients are mostly in the middle and late stages when they are diagnosed with PC, accompanied by proximal infiltration or distant metastasis [9]. Therefore, exploring specific molecular targets that control the development of PC and elucidating its molecular mechanism has important theoretical guiding significance as well as clinical application value for improving early diagnosis and clinical treatment of PC [9] [10].

EPS8L3 is involved in the malignant development of various tumors. Studies have found that EPS8L3 promotes the proliferative ability and metastasis of HCC by regulating EGFR dimerization and internalization [16]. In addition, EPS8L3 up-regulation is related to the occurrence and poor prognosis of HCC [17]. However, little has been reported about the action of EPS8L3 in the development of PC. Here, we aimed to clarify its role and molecular mechanism in PC, which may have signality for the diagnosis and treatment of PC. In this research, qRT-PCR results found that the EPS8L3 level was significantly higher in the PC tumor tissues than that of adjacent ones, and it was in positive correlation with the incidence of metastasis in PC patients. PC is one of the malignant tumors with extremely high malignancy, very rapid pathological process, and abnormally strong proliferative ability and metastasis [18] [19]. Functional studies such as the CCK-8 and Transwell experimentations indicated that EPS8L3 can promote the proliferative ability and metastasis of PC cells, proposing that EPS8L3 may be a key factor in predicting the malignant development of PC.

The bioinformatics predicted that EPS8L3 sequence containing the GSK3B binding site, and dual luciferase reporter gene assay verified that EPS8L3 could be directly bind to GSK3B. GSK3 is a serine/threonine protein kinase and one of the ratelimiting enzymes of glycogen synthase kinase. There are two main subtypes of GSK3 in mammals: GSK3A and GSK3B, with relative molecular weight of 51 kd and 47 kd, respectively. Both of them have similar structures and substrates but different functions [20] [21]. In the early stage, it has been reported that GSK3B is participated in the malignant development of PC [22] [23]. Here, the Western Blot results revealed that knockdown of EPS8L3 can markedly reduce the GSK3B expression in PC cells; and the recovery experimentations also suggested that overexpression of GSK3B can reversal the prohibitive effect of silencing EPS8L3 on the proliferative ability and metastasis of PC cells.

## Conclusion

Taken together, the present study demonstrates that EPS8L3 promotes the development of PC by regulating GSK3B, and that EPS8L3 can be used as a biomarker for early diagnosis and treatment of PC.

## Dodatak

### Conflict of interest statement

All the authors declare that they have no conflict of interest in this work.
